# Pulsed field ablation of a persistent left superior vena cava triggering atrial fibrillation: A case report

**DOI:** 10.1016/j.hrcr.2026.01.025

**Published:** 2026-02-10

**Authors:** Baudouin Koenig, Mathieu Schaaf, Babé Bakouboula

**Affiliations:** 1 Institut Cardiovasculaire de Strasbourg, Clinique Rhéna, Strasbourg, France; 2 Division of Cardiovascular Medicine, Nouvel Hôpital Civil, Strasbourg University Hospital, Strasbourg, France

**Keywords:** Atrial fibrillation, Pulsed field ablation, Congenital anomalies, Safety, Left Superior Vena Cava, Spatiotemporal dispersion

**Key Teaching Points**

• Pulsed field ablation (PFA) represents a novel and effective modality for targeting large or anatomically complex structures.
• PFA is also safe, and potential complications (ie, coronary spasm) can be managed if anticipated.
• Clinicians should maintain a high index of suspicion for underdiagnosed congenital cardiac anomalies as potential drivers of persistent atrial fibrillation (AF), particularly when imaging modalities may fail to exclude them.
• Artificial intelligence-guided spatiotemporal dispersion mapping is useful and effective in order to treat correctly persistent AF in complex cases.

## Introduction

We report the case of a 55-year-old Caucasian man undergoing a third catheter ablation procedure for atrial fibrillation (AF). During the intervention, a previously undiagnosed persistent left superior vena cava (LSVC) appeared to contribute to the maintenance of AF. Given its anatomical characteristics, electrical isolation of the LSVC was performed using pulsed field ablation (PFA) rather than conventional radiofrequency (RF) ablation. PFA seems to be a novel, effective, and safe tool, particularly for targeting larger anatomical structures.

### Case report

#### History of presentation and medical history

A 55-year-old Caucasian man underwent a third catheter ablation for symptomatic, drug-refractory persistent AF. Initial ablation was performed in 2010 and involved pulmonary vein isolation (PVI) and ablation of complex fractionated atrial electrograms (CFAEs) in the left atrium and right atrial septum. Following recurrence of AF, a second procedure was performed in the left atrium in April 2024 leading to a larger PVI, the creation of a posterior box, and cavotricuspid isthmus ablation.

#### Investigations and management

Owing to ongoing symptomatic recurrence (September 2024), a third procedure was scheduled on February 25, 2025, using the EnSite™ X 3D mapping system (Abbott).

**Figure 1:**
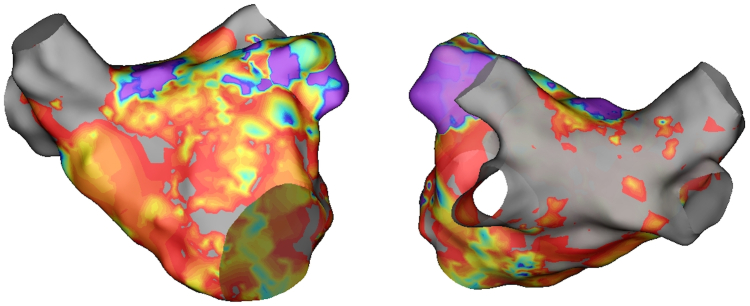
Voltage mapping in atrial fibrillation of the left atrium performed by HD GRID catheter (0.1 mV–0.5 mV). Only the insertion of the Bachmann bundle and the left atrial appendage are highly-volted. Pulmonary veins and posterior wall are isolated. (anterior view on the left and posterior view on the right).

Voltage mapping of the left atrium revealed very low-voltage areas throughout, with 4 isolated pulmonary veins and a disconnected posterior box. Only the anterior left atrial roof (Bachmann’s bundle insertion) and the left atrial appendage exhibited normal voltage (Figure 1).

**Figure 2:**
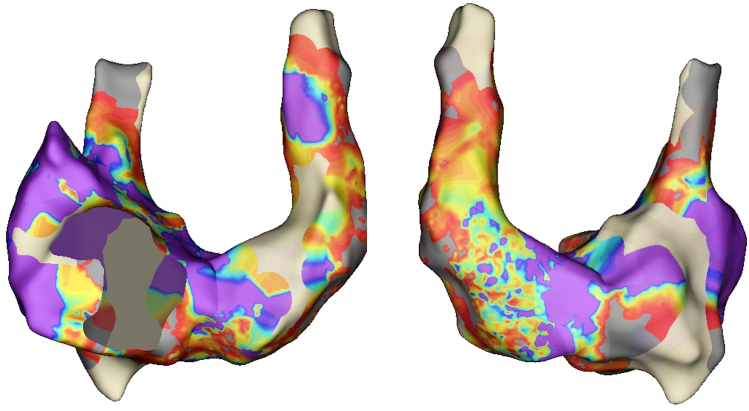
Voltage mapping in atrial fibrillation of the right atrium and the persistent left superior vena cava performed by HD GRID catheter (0.1 mV–0.5 mV). LSVC is low volted, meanwhile the right atrium is normally volted. (anterior view on the left, posterior view on the right). LSVC = left superior vena cava.

Subsequent right atrial voltage mapping unexpectedly revealed a persistent LSVC, which had not been identified during prior procedures. Although the right atrium exhibited normal voltage, the LSVC displayed low-voltage signals (Figure 2).

**Figure 3:**
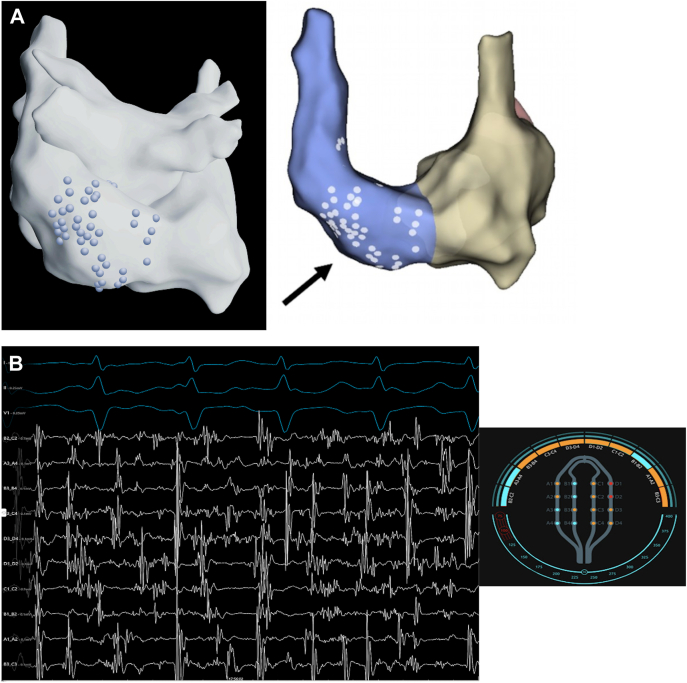
Spatio-temporal-dispersion signals on the posterior wall of the LSVC. **A:** Zones of interest marked by Volta AF-Xplorer (Posterior view: *blue dots* on the left image by Volta AF-Xplorer interface, *white dots* on the right image by EnSite X) **B:** Spatio-temporal-dispersion signals on the electrophysiology bay detected by HD-GRID using the VOLTAPLEX Visualizer. Orange color activation on the Volta AF-Xplorer interface confirms spatio-temporal-dispersion. AF = atrial fibrillation; LSVC = left superior vena cava.

The Volta AF-Xplorer™ system, an artificial intelligence-based tool for identifying relevant electrograms during AF ablation, was employed.^1^ Spatio–temporal-dispersion signals were localized on the posterior wall of the LSVC (Figure 3), whereas no such signals were detected in the left atrium or in the right atrium.

**Figure 4:**
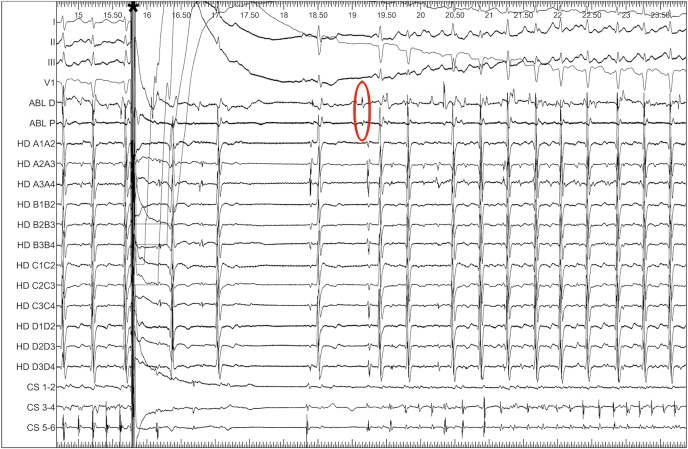
Recurrence of AF after external electrical shock (∗). Earliest signal (*red circle*) detected by the ablation catheter, positioned in the LSVC (earliest atrial signal on the ABL D lead, earlier than the signal in the coronary sinus and on HD GRID). AF = atrial fibrillation; LSVC = left superior vena cava.

External electrical cardioversion briefly restored sinus rhythm, but AF recurred rapidly. Importantly, the primo-activation, obtained by the RF ablation catheter (TactiCath, Abbott) located in the LSVC, preceded left atrial activation obtained by the mapping catheter (HD Grid, Abbott), suggesting its role in AF initiation (Figure 4).

**Figure 5:**
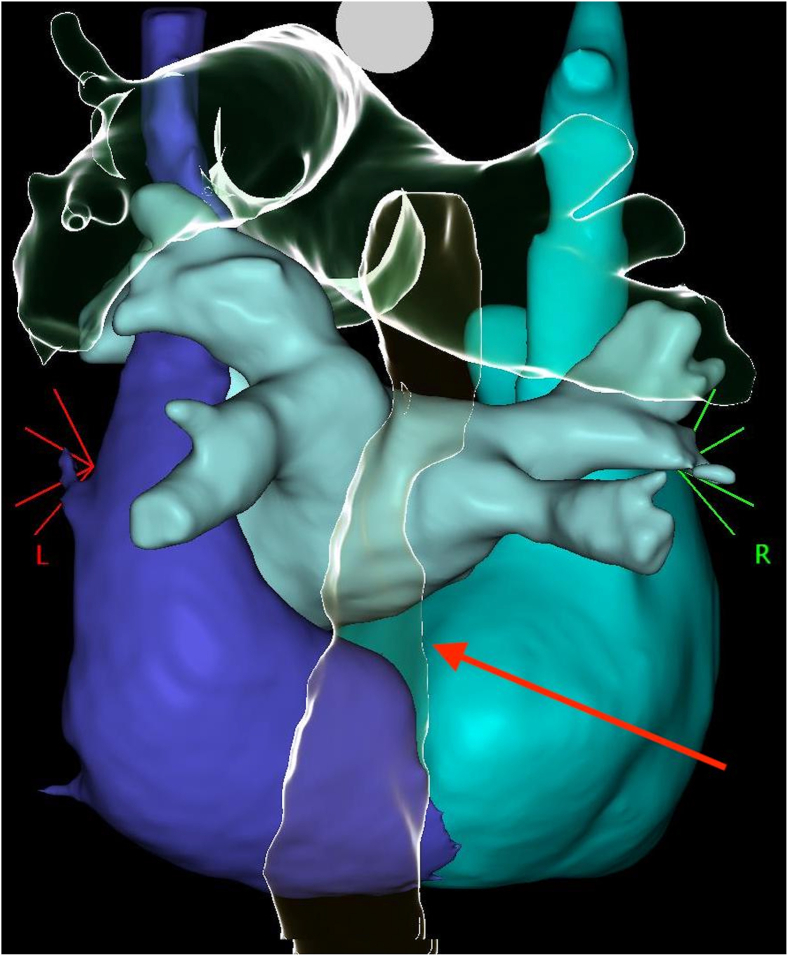
Posterior view of the volume-reconstruction of the atria based on computed-tomography, showing the localization of the esophagus (transparent structure indicated by the *red arrow*). The esophagus crosses the LSVC (*deep blue*) exactly on its junction with the right atrium (*turquoise*), which is the target zone for ablation. LSVC = left superior vena cava.

Complete LSVC isolation via RF was deemed infeasible because of its large ostial dimensions (38 mm [supero-inferior] by 41 mm [antero-posterior]) and its proximity to the esophagus, confirmed by the preprocedural computed tomography (CT)-scan, which posed a risk of thermal injury (Figure 5).

As an alternative, a subsequent procedure using PFA was planned on March 24, 2025. External electrical cardioversion restored sinus rhythm, allowing to perform an electrical activation and voltage map in sinus rhythm. It confirmed low-voltage areas in LA, and the activation of the LAA from the Bachmann. The upper part of the LSVC was activated by the LAA and the distal Bachmann bundle, whereas the lower part of the LSVC was activated by the RA, the coronary sinus, and the LA posterior wall.

**Figure 6:**
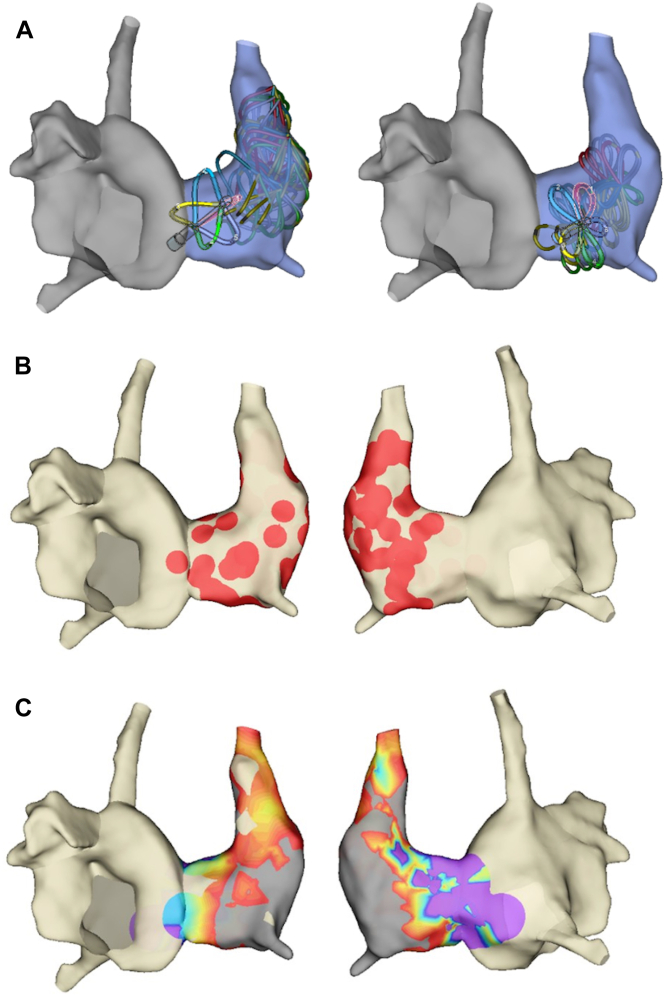
PFA ablation in the LSVC. **A:** FARAWAVE ablation positions localized in the distal part of the LSVC, in both conformations (basket on the left, and flower on the right). **B:** Localization of the ablation zone (*red dots*) performed by FARAWAVE in the LSVC (anterior view on the left and posterior view on the right). **C:** Voltage map of the LSVC after ablation, performed in sinus rhythm performed by a FARAWAVE pentaspline catheter (0.5 mV–1.5 mV) (anterior view on the left and posterior view on the right). No or very low-voltage in the LSVC. LSVC = left superior vena cava.

PFA was performed at the LSVC–right atrial junction using the FARAPULSE™ system (Boston Scientific) with EnSite™ X guidance, under NavX modality. The patient was in AF at the beginning of the procedure. Ablation was performed with a 35 mm-pentaspline-catheter (FARAWAVE™, Boston Scientific) targeting areas previously identified by the Volta system (Figure 6A and B). A total of 28 applications (18 in basket mode, 10 in flower mode) were delivered, leading to restoration of sinus rhythm. Voltage map of the LSVC in sinus rhythm showed no or very low-voltage in the LSVC (Figure 6C).

To reduce the theoretical risk of coronary spasm, intracoronary 2 mg nitroglycerin-bolus in the LAD (left anterior descending coronary artery) and subsequent continuous peripheral intravenous nitroglycerin-infusion (1 mg/mL per min) were given during the entire PFA (8 min). To assess the absence of coronary spasm, especially involving the circumflex artery, coronary angiography was performed before, during, and after PFA. No coronary spasm was observed, and the coronary anatomy was found to be right-dominant.

#### Outcome and follow-up

No complication occurred and no recurrence of AF was observed during the 5 months follow-up.

## Discussion

This case highlights the potential arrhythmogenic role of a persistent LSVC in patients with persistent AF. A persistent LSVC is a rare congenital anomaly, resulting from the failure of the left anterior cardinal vein to regress during embryogenesis. It has a reported prevalence of 0.2%–3% in the general population.

Although frequently asymptomatic, a persistent LSVC may be associated with persistent AF. This association could be explained by: (1) its common embryologic origin with the vein of Marshall, a known target for the treatment of persistent atrial fibrillation, presenting myocardial connections and arrhythmogenic foci; (2) its frequent association with other congenital cardiac anomalies; and (3) its anatomic connections to both atria.

The presence of LSVC as a trigger or driver for AF has been previously reported.^2^^,^^3^

Clinicians face 3 main challenges with persistent LSVC: **(1) Diagnostic Oversight**: In our case, LSVC was missed despite 2 prior ablation procedures and cardiac CT. This is often because of the lack of symptoms and the limitations of routine imaging, which usually includes only arterial-phase studies. Accurate diagnosis typically requires venous-phase imaging or left-arm contrast injection. **(2) Therapeutic Complexity**: Owing to its size and anatomic connections to both atria, achieving complete electrical isolation of the LSVC using point-by-point RF ablation is challenging. Cryoablation of LSVC has also been reported to date.^4^ **(3) Iatrogenic risk:** The posterior course of the LSVC, in close relation to the esophagus, significantly increases the risk of thermal injury when using RF ablation. PFA is an interesting alternative to reduce this risk, but the occurrence of coronary vasospasm of the circumflex artery with this technique should be considered. Our case report demonstrates the safety of the nitroglycerin-infusion protocol previously reported by Malyshev et al.^5^

PFA is a promising technique that uses nonthermal energy to achieve myocardial ablation, offering safety advantages such as the absence of esophageal injury and pulmonary vein stenosis.^6^ As demonstrated in this case and previously by Castiglione et al.^7^ and Gupta et al.,^8^ PFA may be both effective and safe for ablation in patients with congenital venous anomalies.

In our patient, PFA enabled successful LSVC isolation with no complications, providing strong evidence for its use in similar settings.

## Conclusion

PFA appears to be a safe and effective strategy for the electrical isolation of a persistent LSVC involved in AF, particularly when anatomical considerations limit the use of conventional RF ablation.

This case underscores the importance of considering LSVC as a potential source of AF and the need for appropriate diagnostic imaging to avoid underdiagnosis of this arrhythmogenic structure.

## Disclosures

All authors declare that they have no competing interest.

## References

[1] Deisenhofer I., Albenque J.P., Busch S. (2025). Artificial intelligence for individualized treatment of persistent atrial fibrillation: a randomized controlled trial. Nat Med.

[2] Hsu L.F., Jais P., Keane D. (2004). Atrial fibrillation originating from persistent left superior vena cava. Circulation.

[3] Kim Y.G., Han S., Choi J.I. (2019). Impact of persistent left superior vena cava on radiofrequency catheter ablation in patients with atrial fibrillation. Europace.

[4] Schneider M.A., Schade A., Koller M.L., Schumacher B. (2009). Cryoballoon ablation of paroxysmal atrial fibrillation within the dilated coronary sinus in a case of persistent left superior vena cava. Europace.

[5] Malyshev Y., Neuzil P., Petru J. (2024). Nitroglycerin to ameliorate coronary artery spasm during focal pulsed-field ablation for atrial fibrillation. JACC Clin Electrophysiol.

[6] Metzner A., Fiala M., Vijgen J. (2024). Long-term outcomes of the pentaspline pulsed-field ablation catheter for the treatment of paroxysmal atrial fibrillation: results of the prospective, multicentre FARA-Freedom Study. Europace.

[7] Castiglione A., Kuffer T., Grani C., Servatius H., Reichlin T., Roten L. (2023). Pulsed-field-ablation for the treatment of atrial fibrillation in patients with congenital anomalies of cardiac veins. J Cardiovasc Electrophysiol.

[8] Gupta A., Sundhu M., Reddy M., Sheldon S.H., Noheria A. (2024). Sequential isolation of persistent left superior vena cava and right superior vena cava using pulsed-field ablation with a pentaspline catheter for recurrent persistent atrial fibrillation. J Innov Card Rhythm Manag.

